# 

**Published:** 1890-05-31

**Authors:** 


### SPECIAL HOSPITAL SUNDAY NUMBER.

SATURDAY, MAY 31st, 1890.

**A Christian City** . . . . . . . . . . 115
**The Year's Work of the Medical Charities of London** 116
**The Story of the Hospitals**—Hospitals and Sick Children—
Chapter I. Introductory . . . . . . . . 117
„ II. A Catastrophe . . . . . . . . 118
„ III. Hospital Life . . . . . . . . 119
Chapter IV. Nurses and Patients . . . . . . 121
„ V. At the West-End Home . . . . . . 123
„ VI. Existence at the East-End . . . . . 125
„ VII. Tired Out . . . . . . . . 126
„ VIII. Something to Ponder Over . . . . . 127
„ X. The End of the Matter . . . . . . 129

### EXTRA SUPPLEMENT—THE NURSING MIRROR.

**En Passant.**—During the Plague—Registration of Midwives—A Home of Rest—Short Items—Hammersmith Nursing Association . . . . . . . . xxxiii
**The History and Capabilities of Herbal Simples.** By W. T. Fernie, M.D. X.—Broom . . . . . xxxiv
**American Training Schools for Nurses** . . . xxxiv
**A Letter from South Africa** . . . . . . . xxxv
**Everybody's Opinion.**—The Cure of Drunkards . . xxxvi
**Appointments** . . . . . . . . . . xxxvi
**Notes and Queries** . . . . . . . . . xxxvi
**Vacancies** . . . . . . . . . . xxxvi
**Amusements and Relaxation** . . . . . . xxxvi

